# Poly(ε-caprolactone) (PCL) Hollow Nanoparticles with Surface Sealability and On-Demand Pore Generability for Easy Loading and NIR Light-Triggered Release of Drug

**DOI:** 10.3390/pharmaceutics11100528

**Published:** 2019-10-13

**Authors:** Ju Hyang Park, Da In Kim, Sang Gi Hong, Hojun Seo, Jongbok Kim, Geon Dae Moon, Dong Choon Hyun

**Affiliations:** 1 Department of Polymer Science and Engineering, Kyungpook National University, Daegu 41566, Korea; pjh99279@naver.com (J.H.P.); dada7230@naver.com (D.I.K.); abio2007@naver.com (S.G.H.); dark911205@gmail.com (H.S.); 2 Dongnam Regional Division, Korea Institute of Industrial Technology, Busan 46938, Korea; 3 Department of Materials Science and Engineering, Kumoh National Institute of Technology, Gumi, Gyeongbuk 39177, Korea; jb1956k@gmail.com

**Keywords:** porous particle, poly(ε-caprolactone), hollow particle, NIR-triggered drug release, anticancer activity

## Abstract

A new system for the easy loading and NIR light-triggered release of drugs is introduced. It consists of poly(ε-caprolactone) (PCL) hollow nanoparticles with surface openings containing a biodegradable fatty acid with phase-change ability and a biocompatible photothermal agent. These openings, which can enhance the connectivity between the interior and the exterior, enable the easy loading of drug molecules into the interior voids, and their successive sealing ensures a stable encapsulation of the drug. Upon exposure to an external NIR light irradiation, the photothermal agent generates heat that raises the local temperature of the hollow particles above the melting point of the fatty acid, leading to the formation of nanopores on their shells, and consequently, the instant release of the encapsulated drug molecules through the pores. The synergistic activity of the hyperthermia effect from the photothermal agent and the NIR-triggered release of the drug molecules results in noticeable anticancer efficacy.

## 1. Introduction 

Over the past several decades, remarkable progress has been made in the fabrication of polymer nanoparticles (NPs) with a diameter in the range of 1 to 1000 nm for applications in the controlled release and delivery of drugs [1,2]. In particular, polymer hollow NPs, which are characterized by interior voids and outer shells, have sparked a growing interest because their hollow interiors can highly encapsulate bioactive drugs, and their shells can efficiently protect the bioactivity of encapsulated drugs from deactivation [3,4]. Furthermore, the shells function as diffusion barriers against the undesired noticeable release of drugs at the initial stage and allow their linear release over time, which is called zero-order release [5,6,7].

Although this release pattern is beneficial for chemotherapy, many clinical situations require the drugs to be released on-demand rather than continuously [8,9,10]. This has led to the development of stimuli-responsive polymer hollow NPs, which allow a controlled release of drugs in response to environmental stimuli [11,12,13,14,15]. The stimuli responsiveness is achieved by including functional monomers or inorganic components in the hollow NPs [4]. As an example, Yang and coworkers demonstrated the fabrication of double-walled hollow nanoparticles with uniform size through the synthesis of silica/polymer hybrids by alternating sol–gel and distillation–precipitation polymerization processes, followed by the selective removal of the silica component. The resulting NPs were composed of a poly(methacrylic acid) (PMAA) inner shell and a poly(*N*-isopropylacrylamide) (PNIPAM) outer shell with independent sensitivity to external pH and temperature [16]. In a similar way, poly(*N*,*N*′-methylene bisacrylamide-*co*-methacrylic acid) (P(MBAAm-*co*-MAA)) hollow NPs were prepared from PMAA/P(MBAAm-*co*-MAA) core-shell particles through the selective removal of the non-crosslinked PMAA core in ethanol. The release of the anticancer drug molecules encapsulated into the core was strongly dependent on pH [17]. Chiu and coworkers used hydrogel hollow NPs incorporated with superparamagnetic iron oxide NPs to accelerate the anticancer drug release in response to both pH and temperature [15]. Recently, polymer hollow NPs responsive to typical physiological stimuli such as pH and redox potential were also fabricated via the self-assembling of a novel amphiphilic diblock copolymer, allowing the release of tetraphenylporphyrin tetrasulfonic acid hydrate for photodynamic therapy [18]. However, most of these systems consist of non-biocompatible, non-biodegradable components, which have hindered their practical use in biomedical applications. Moreover, since the closed shells of these particles make it difficult to load drug molecules in the interior voids through diffusion, their drug-loading capacity is very low, and the encapsulation of macromolecular drugs is limited. 

In this study, we introduce biocompatible and biodegradable polymer hollow NPs for the easy loading and near infrared (NIR) light-triggered release of drugs. The fabrication of the hollow NPs involves the use of soft lithography to pattern a composite film made of poly(ε-caprolactone) (PCL), a naturally occurring biodegradable fatty acid (FA) with phase-change ability, and the Food and Drug Administration (FDA)-approved, biocompatible indocyanine green (ICG) as a photothermal agent to an array of discrete rings, followed by their transformation into spherical hollow NPs with surface openings. The openings, which can enhance the connectivity between the interior and the exterior, enable the easy loading of drug molecules into the interior voids, and their successive sealing ensures a stable encapsulation of the drug. Upon exposure to an external NIR source, the photothermal agent entrapped into the shells of these hollow NPs can generate heat that raises the local temperature of the NPs above the melting point of the constituent FA, leading to the formation of nanopores on their shells, and consequently, the instant release of the encapsulated drug molecules through the pores. Thus, a noticeable anticancer activity can be achieved through the combination of the hyperthermia effect from the photothermal agent and the NIR light-triggered release of the drug molecules. 

## 2. Materials and Methods

### 2.1. Materials

PCL (M_n_ ≈ 10 kDa), bovine serum albumin (BSA), BSA labeled with fluorescein isothiocyanate (FITC-BSA), lauric acid (LA, 98%), stearic acid (SA, 98.5%), poly(vinyl pyrrolidone) (PVP, M_w_ ≈ 55 kDa), phosphate buffer saline (PBS, pH 7.4), 4′,6-diamidino-2-phenylindole (DAPI), and water-soluble tetrazolium salt-1 (WST-1) solution were purchased from Sigma-Aldrich (St. Louis, MO, USA). Toluene, chloroform, 1,4-dioxane, and 2,2,2-trifluoroethanol (TFE) were obtained from Duksan Chemical (Daegu, Korea). Doxorubicin hydrochloride (DOX) and ICG were provided by Tocris (Bristol, United Kingdom) and Acros (Morris, NJ, USA), respectively. The Sylgard 184 elastomer kit obtained from Dow Corning (Midland, MI, USA) was used to fabricate a poly(dimethyl siloxane) (PDMS) mold with an array of cylindrical wells (1 μm in diameter, 0.3 μm in height, and separated by 0.6 μm) on its surface, as previously reported [5]. Human breast cancer SK-BR-3 and normal human dermal fibroblast (NHDF) cells were purchased from Korean Cell Line Banks (Seoul, Korea). Deionized (DI) water (18.2 MΩ) produced by an ultra water purification system (ROMAX, Human Science, Hanam, Korea) was used as a dispersion medium for the hollow NPs.

### 2.2. Fabrication of FA-Incorporated PCL Hollow NPs 

A eutectic mixture of FAs consisting of LA and SA was prepared, as previously reported [19], and then dissolved along with PCL in a co-solvent of toluene and chloroform (3:1, *w*/*w*). The concentration of PCL in the mixed solution was fixed at 0.2 wt%, while the concentration of the FA mixture varied from 0.02 to 0.04 wt%. The resultant solutions were spin-coated at 4000 rpm for 30 s on a PVP-coated substrate with a dimension of 5 cm × 5 cm, which were prepared using a previously reported procedure [5]. The patterned PDMS mold with a dimension of 5 cm × 5 cm was brought into conformal contact with this composite layer, and then thermally annealed in a vacuum oven that was pre-heated to 80 °C. After 10 min, the sample was taken out from the oven, followed by cooling down in air. Upon carefully peeling off the PDMS mold from the substrate, an array of the composite rings consisting of the FA mixture and PCL was generated. These rings were released from the substrate by dissolving the PVP layer with 4 mL of an aqueous PVP solution (5 wt%), followed by the addition of toluene (20 μL). After 1 h, the resultant suspension was added drop-wise into a 20-mL vial pre-containing 20 mL of liquid nitrogen (LN_2_). Next, the frozen sample was placed in a freeze-drier for 24 h to generate the desired hollow NPs with openings on their shells. In order to endow the hollow NPs with NIR-light sensitivity, commercial hydrophilic ICG was converted into hydrophobic ICG-tetrabutylamine according to a previously reported procedure [20]. A composite layer made of hydrophobic ICG, eutectic FA mixture, and PCL, which was prepared by spin-coating its solution, was used to make the NIR-sensitive hollow NPs with surface openings. The concentration of ICG in the solution ranged between 0.0004–0.0015 wt%. 

### 2.3. Drug Encapsulation 

For the encapsulation of DOX, the dried hollow NPs (0.4 mg) were dispersed in its aqueous solution (2 mL) with a concentration of 25 mg/mL. After magnetic stirring at 100 rpm for 30 min, 20 μL of toluene was added to the mixture solution, which could completely seal the openings in 30 min at room temperature. The non-specifically adsorbed drug molecules were removed by two rounds of repeated centrifugation and washing with DI water. For the encapsulation of BSA, we used an aqueous solution of BSA (200 mg/mL) and added 35 μL of 1,4-dioxane, instead of toluene, to the system. FITC-BSA was also loaded using its aqueous solution with a concentration of 25 mg/mL. 

### 2.4. Determination of Composition of Hollow NPs

The amount of FA mixture loaded in the hollow NPs was evaluated by comparing the weights of the NPs before and after the removal of the mixture using a microbalance (XS105, Mettler Toledo, Greifensee, Switzerland) with a precision of ~0.01 mg. For the evaluation, the hollow NPs (50 mg) with the inclusion of FA mixture were mixed with water (5 mL) at 40 °C, followed by centrifugation at 12,000 rpm for 10 min. After removing the supernatant, the sample was stored in vacuum for 24 h to completely remove residual water. The amounts of loaded ICG and DOX were checked by dissolving 10 mg of the NPs loaded with them in 2 mL of TFE, followed by analyzing 1 mL of the solution via ultraviolet–visible (UV-Vis) spectrophotometry (T60, PG Instrument, Leicestershire, UK), as previously reported [21]. The amount of loaded DOX was determined from the UV-Vis absorbance at 490 nm using a calibration curve constructed from a series of drug solutions in TFE. In a similar way, we determined the amount of loaded ICG from the UV-Vis absorbance at 780 nm using a calibration curve obtained from a series of ICG solutions in TFE. The amount of encapsulated FITC-BSA was indirectly derived from that of the not encapsulated portion, which was determined by centrifuging the hollow NPs after the encapsulation process and collecting 1 mL of the colloid-free solution, as reported in a previous literature [22].

### 2.5. Photothermal Ability 

The ICG-loaded hollow NPs (5 mg) were dispersed in 5 mL of a PBS solution, followed by storage at 37 °C for 6 h. Then, the sample was exposed to 0.7 W/cm^2^ NIR light (wavelength of 808 nm) generated by fiber-coupled NIR light-emitting diodes (LVI Technology, Yongin, Korea). The change in the temperature of the sample was measured using a fiber optic thermometry unit (LuxtronMD600) connected to a computer. 

### 2.6. In Vitro Release Test

In order to obtain the release profiles of drugs for 12 and 18 h, the NPs (5 mg) encapsulated with DOX or FITC-BSA were dispersed in 5 mL of a PBS solution contained in a centrifuge tube, which was then tightly sealed and placed in a water bath at 37 °C under mild agitation. At different time intervals, the suspension was centrifuged (12,000 rpm, 10 min), followed by taking out 1 mL of the colloid-free solution and then adding 1 mL of fresh buffer under mechanical agitation to re-suspend the NPs without any aggregation. The amount of released DOX was determined from the UV-Vis absorbance at 478 nm using a calibration curve constructed from a series of DOX solutions in PBS buffer, as previously reported [21]. The amount of released FITC-BSA was evaluated from a calibration curve of the fluorescence intensity for solutions with known concentrations of FITC-BSA obtained using a microplate reader (EL800, BIO-TEK, Winooski, VT, USA), as reported in previous literature [22]. In order to obtain the drug release profiles in response to NIR light irradiation, the hollow NPs were treated once with NIR light (0.7 W/cm^2^) for 5 min at the beginning of the release test. The percentage of drug release was calculated by dividing the accumulative amount of drug released at a given time by the initial amount loaded. 

### 2.7. Cell Viability Test

The viabilities of SK-BR3 and NHDF cells against the hydrophobic ICG-tetrabutylamine and the NIR light irradiation were evaluated using the WST-1 assay, as previously reported [21]. Briefly, each type of the cells was seeded at a density of 20,000 cells/well in a 24-well cell culture plate for 24 h. In order to investigate the cytotoxicity of the hydrophobic ICG to the cells, the medium was replaced with 1 mL of fresh medium, followed by introducing a hydrophobic ICG solution (10 μL) in dimethyl sulfoxide with a certain concentration. Then, the cells were incubated at 37 °C for 48 h to conduct the WST-1 assay. Similarly, the cytotoxicity of NIR light irradiation (808 nm) was examined. After replacement with fresh medium (1 mL), the cells were treated with 0.7 W/cm^2^ NIR light for 2–10 min, and then incubated for an additional 48 h. The cytotoxicity of the PCL hollow NPs entrapping the FA mixture in their shells (PCL/FA NPs) was investigated by incubating each type of the cells cultured through the aforementioned procedure with 1 mL of fresh medium containing these NPs with a certain concentration for 4 h, followed by washing with PBS solution and further incubation with fresh medium for 44 h. In a similar way, we evaluated the anticancer activities of the PCL hollow NPs encapsulating DOX (PCL/DOX NPs), the DOX-encapsulated hollow NPs entrapping the FA mixture in their shells (PCL/FA/DOX NPs), the PCL hollow NPs entrapping both the FA mixture and ICG in the shells (PCL/FA/ICG NPs), and the DOX-encapsulated hollow NPs entrapping the FA mixture and ICG (PCL/FA/ICG/DOX NPs). After incubation with fresh medium (1 mL) containing the NPs (1 mg) for 4 h, washing with PBS solution, and NIR irradiation (0.7 W/cm^2^, 5 min) in fresh medium, the SK-BR3 cells were further incubated with fresh medium at 37 °C for 44 h for the WST-1 assay.

### 2.8. Confocal Laser Scanning Microscopy

The SK-BR3 cells were seeded at a density of 10,000 cells/well on a glass cover slip placed in a 12-well culture plate and incubated for 24 h. Then, the medium was replaced with fresh medium containing the DOX-loaded hollow NPs (0.5 mg/mL) or free DOX, followed by incubation for 4 h at 37 °C. The cells were exposed to NIR light (0.7 W/cm^2^, 8 min) and further incubated at 37 °C. After 3 h, they were washed with PBS solution and fixed with a 4% paraformaldehyde solution. Next, their nuclei were counterstained with DAPI. The intracellular localization of DOX was observed with a confocal laser scanning (CLS) microscope (LSM700, Carl Zeiss, Oberkochen, Germany).

### 2.9. Characterization

Transmission electron microscopy (TEM) images were acquired using an HT-7700 microscope (Hitachi, Tokyo, Japan) operated at 75 kV to investigate the morphologies of the fabricated NPs. The TEM images of the hollow NPs were used to measure their outer and inner diameters. Scanning electron microscopy (SEM) images were acquired using an SU-8220 microscope (Hitachi, Tokyo, Japan) operated at an accelerating voltage of 3 kV to determine the outer diameters of the hollow NPs and the sizes of their openings. The mean values were calculated using the results of over 100 particles randomly selected from each sample, and the standard deviation was represented by the errors. The ζ-potential and size distribution of the NPs were investigated using a Zetasizer Nano-ZS dynamic light-scattering (DLS) analyzer (Malvern Instruments, Worcestershire, UK). In order to demonstrate the size distribution, the standard deviation was represented by the errors. The inclusion of the FA mixture in the NPs was confirmed using differential scanning calorimetry (DSC) (Q2000, TA Instrument, New Castle, DE, USA). All the measurements were performed in the range of 20 to 230 °C at a scanning rate of 2 °C/min. 

## 3. Results and Discussion

**Figure 1:**
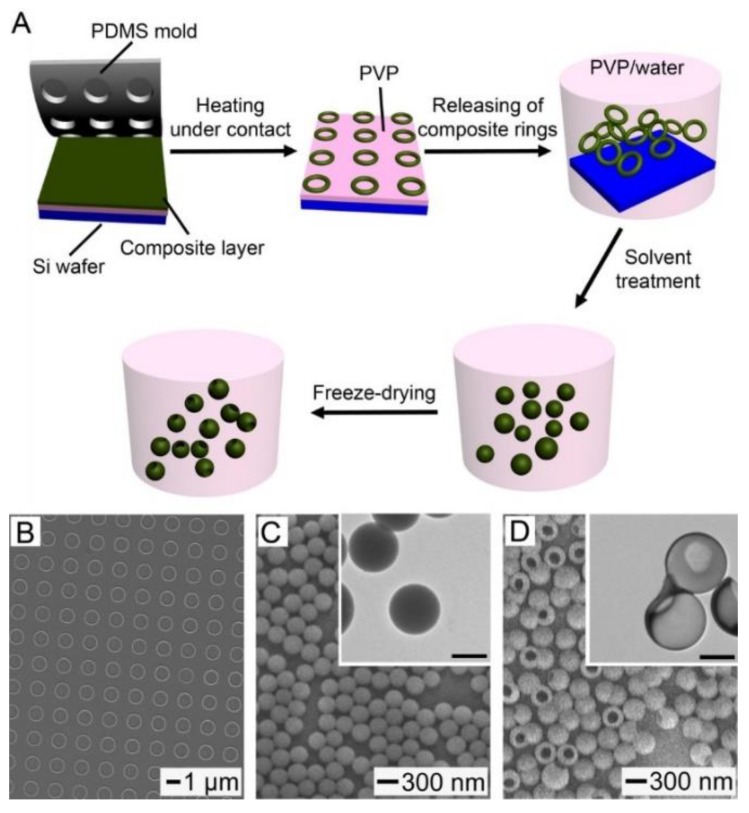
(**A**) Schematic illustration of a procedure for fabricating the hollow nanoparticles (NPs) made of poly(ε-caprolactone) (PCL) and a eutectic fatty acid (FA) mixture. (**B**) SEM image of an array of the composite rings generated on the PVP layer. (**C**,**D**) SEM and TEM (inset) images of: (**C**) The solid nanospheres obtained by the solvent treatment for 1 h and (**D**) the hollow NPs with openings on their shells. The scale bars in the insets represent 200 nm.

In order to fabricate biocompatible and biodegradable hollow NPs for the easy loading and NIR light-triggered release of drugs, we chose to work with PCL and FAs due to their attractive features. Along with excellent biocompatibility and biodegradability, PCL has a slow degradation rate that is advantageous in the stable encapsulation of payloads without their undesired release [23]. FAs are naturally occurring biocompatible and biodegradable materials that are capable of reversible solid–liquid transitions in response to small variations in temperature [10,24]. In particular, the eutectic mixture made of LA and SA has increasingly found biomedical applications because its melting point (i.e., 38–40 °C) is close to the physiological temperature of human bodies [25,26,27,28]. Figure 1A schematizes the fabrication procedure for the hollow NPs consisting of PCL and the eutectic FA mixture. Four major steps are involved: the generation of discrete PCL/FA composite rings on top of a PVP layer; the release of the rings into an aqueous solution of PVP; the conversion of the rings into solid nanospheres through solvent treatment; and their transformation into hollow NPs with openings on their shells via freeze-drying.

Figure 1B shows an SEM image of an array of the composite rings consisting of PCL and the eutectic mixture of LA and SA, supported on the PVP-coated substrate. The initial PCL/FA composite layer was 4.1 nm in thickness, which was prepared by spin-coating its solution with 0.02 wt% of the FA mixture and 0.2 wt% of PCL. The FA mixture and PCL were melted and dewetted by heating up to 80 °C, being transformed to the droplets that were randomly distributed on the surface of PVP (Figure S1, Supplementary Materials). The recessed regions on the PDMS mold guided this dewetting process, since the capillary force pushed the melted composite into the cylindrical wells on the mold [5], leading to the generation of an array of PCL/FA composite rings. The ring shape resulted from the production of composite meniscus along the PDMS walls, which is a signature mark of the capillary rise [29].

The generated rings were released into the aqueous solution of PVP, followed by addition of a small amount of toluene. Figure 1C shows an SEM image of the resultant sample after the release, solvent treatment for 1 h, and its evaporation in air. Upon the addition of toluene, which is highly miscible with PCL and the FA mixture but immiscible with water, the solvent diffused into the rings to have the composite molecules migrated even at room temperature. Thus, the rings were transformed into nanospheres, reducing the contact area with water. The TEM image in the inset demonstrates that these spheres had a solid structure with uniform size. They had a *ξ*-potential of −29.8 mV, and their mean diameter measured from these images was 284 ± 12 nm, which was comparable to the result obtained by DLS (301 ± 18 nm) and the theoretically calculated value (Figure S2, Supplementary Materials). The diameter can be easily controlled by varying the thickness of the composite layer used to generate the discrete rings, because there is a one-to-one correspondence between the solid NP and ring [5]. 

Figure 1D shows an SEM image of the NPs with well-defined openings on their shells, which were fabricated by releasing the composite rings and treating with toluene, followed by freeze-drying in vacuum. Each particle exhibited an opening of ca. 165 nm in diameter on its shell and an increased outer diameter, compared with the solid nanospheres of Figure 1C, of 340 ± 20 nm. A TEM image is shown in the inset, confirming that these NPs were hollow. The formation of the interior voids and openings was because of the strong evaporation flux of toluene [10]. The solvent treatment led to the conversion of the rings into the solid NPs, as shown in Figure 1C, but the solvent was still present in the NPs due to its greater affinity to the composite rather than to water. After freezing into an LN_2_ bath, the NPs was slowly warmed up in vacuum to reach a temperature above the melting point (−95 °C) of the solvent. As driven by the strong evaporation flux of the solvent, the polymer chains and FA molecules moved toward the surface of each particle, leading to the formation of a cavity in the interior and an opening on the shell.

**Figure 2:**
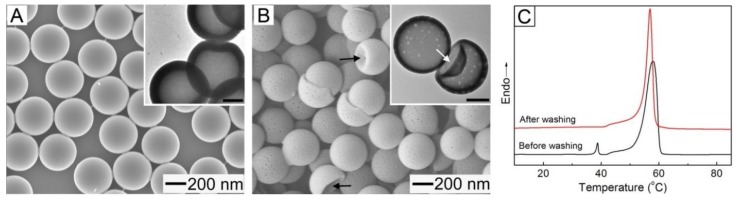
(**A**) SEM and TEM (inset) images of the hollow NPs obtained after suspending the sample of Figure 1D in a mixture of toluene and water for 30 min. The toluene-to-water ratio in volume was 0.01. (**B**) SEM and TEM (inset) images of the resultant sample after washing the particles of (**A**) with water at 40 °C. The scale bars in the insets represent 100 nm. (**C**) Differential scanning calorimetry (DSC) thermograms for the hollow NPs before and after washing with water.

The openings could be completely sealed at room temperature through solvent annealing using a good solvent for the FA mixture and PCL. Figure 2A shows an SEM image of the sample obtained after suspending the NPs of Figure 1D in water, followed by the addition of a small amount of toluene as a plasticizer. The solvent treatment decreased the mean diameter of the resultant particles to 310 ± 15 nm and completely closed the openings on their shells. Furthermore, as shown in the TEM image of the inset, the hollow interiors of the particles were retained after the openings were closed. In order to investigate the driving force of the sealing, we theoretically calculated the interfacial free energy (*E*) for a hollow particle with an open hole on its surface using a simple model (Figure S3, Supplementary Materials). *E* can be expressed by the interfacial tension between the inner surface and water (*γ_inner/water_*), the interfacial tension between the outer surface and water (*γ_outer/water_*), the outer (*d_outer_*) and inner (*d_inner_*) diameters, and the two angles (*θ_inner_* and *θ_outer_*) [30,31]:

$$E=\gamma_{outer/water}\left[\frac{\pi d_{outer}^{2}}{2}(1+\cos\theta_{outer})\right]+\gamma_{inner/water}\left[\frac{\pi d_{inner}^{2}}{2}(1-\cos\theta_{inner})\right]$$

The relation among *d_inner_*, *d_outer_*, *θ_inner_*, and *θ_outer_* is established by considering the diameter of opening (*D_H_*), which has a form of *D_H_* = *d_inner_* sin*θ_inner_* = *d_outer_* sin*θ_outer_* [31,32]. By substituting the values of *d_inner_*, *d_outer_*, and *D_H_* measured by SEM and TEM characterizations into the relation, we obtained the theoretical values of *θ_inner_* and *θ_outer_*. The interfacial tension could be estimated using a harmonic mean equation given as: *γ_surface/water_* = *γ_surface_* + *γ_water_* − 4*γ_surface_^d^γ_water_^d/^*(*γ_surface_^d^* + *γ_water_^d^*) − 4*γ_surface_^p^γ_water_^p/^*(*γ_surface_^p^* + *γ_water_^p^*). By assuming a PVP-coated outer surface and a PCL inner one for the hollow particle, we could obtain *γ_outer/water_* from the surface tensions for water and PVP available in literature [31], while *γ_inner/water_* was calculated using the surface tension for PCL instead of that for PVP [33]. The calculated *E* for a hollow NP with a closed shell (Figure 2A) was calculated to be 5.39 × 10^−15^ J, which was smaller than the value (6.95 × 10^−15^ J) for a NP with a surface opening (Figure 1D). This result suggests that the sealing of the opening was due to the reduction in *E*.

Figure 2B shows an SEM image of the NPs obtained after washing the sample of Figure 2A with water at 40 °C, which is slightly above the melting point of the FA mixture, confirming the generation of nanopores with a diameter of ca. 15 nm on the shell of each particle. The NPs marked by arrows exhibited a deformed structure (with a dimple), which implies that they were still hollow. The TEM image in the inset clearly demonstrates the generation of the nanopores on the shell and the presence of the hollow interior. In order to understand the formation of these pores, we conducted a DSC analysis for the hollow NPs before and after the washing (Figure 2C). The non-washed sample exhibited two sharp peaks at 39 °C and 58 °C that corresponded to the FA mixture and PCL, respectively, but the peak from the FA mixture was not observed for the washed sample. These results indicate that the formation of the nanopores was due to the removal of the FA mixture phase-separated with PCL from the shell. The loading content of the FA mixture in the non-washed NPs (defined as a weight percentage of the mixture relative to the NPs) was 7.3%. 

**Figure 3:**
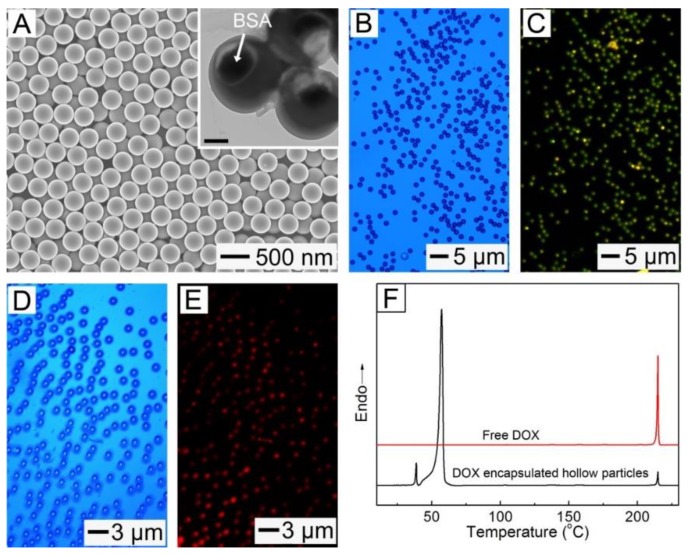
(**A**) SEM and TEM (inset) images of the hollow NPs encapsulating bovine serum albumin (BSA) in their interior voids. The scale bar in the inset represents 100 nm. (**B**,**D**) Optical and (**C**,**E**) fluorescence micrographs of the hollow particles encapsulating: (**B**,**C**) BSA labeled with fluorescein isothiocyanate (FITC-BSA) and (**D**,**E**) doxorubicin hydrochloride (DOX). (**F**) DSC thermograms of the DOX-encapsulated hollow NPs and free DOX.

The formation and sealing of the openings on the shells of the hollow NPs can be advantageous for the quick and stable encapsulation of various types of drugs. In order to demonstrate the efficient encapsulation of macromolecular drugs, the NPs shown in Figure 1D were mixed with an aqueous solution of BSA, followed by solvent annealing for 30 min. We used 1,4-dioxane, instead of toluene, for the annealing because the aqueous dioxane did not cause the denaturation of the protein [7]. The use of 1,4-dioxane resulted in the complete closing of the surface openings, and consequently allowed the stable loading of BSA, as shown in Figure 3A. Figure 3B,C show the optical and fluorescence micrographs of the hollow particles encapsulating FITC-BSA in their interior voids. For an easy optical microscopy observation, the hollow particles with *d_outer_* of 1.2 μm were used (Figure S4, Supplementary Materials). Each particle exhibited a fluorescence due to FITC-BSA (Figure 3C), which confirms the effective encapsulation of the protein in the hollow interiors of all the particles. The amount of BSA encapsulated in a hollow particle is given by: *M_BSA_* = π(4/3)(*d_inner_*/2)^3^·*C_BSA_*, where *C_BSA_* is the concentration of FITC-BSA solution. The mass of a hollow particle encapsulated with the protein is expressed as: *M_Hollow_* = π(4/3)[(*d_outer_*/2)^3^ − (*d_inner_*/2)^3^] *ρ_Hollow_* + *M_BSA_*, where *ρ_Hollow_* is the density of the particle. Based on the values of *d_outer_* (1.2 μm) and *d_inner_* (0.9 μm) obtained from the TEM image in Figure S4 along with the assumption of *ρ_Hollow_* ≈ *ρ_PCL_* = 1.1 g/mL [34], the calculated encapsulation content (*M_BSA_*/*M_Hollow_* × 100) was 1.7%, which was in reasonable agreement with the experimental value of 2.2%. This result suggests that the amount of drug encapsulated in the hollow particles could be readily controlled by varying the concentration of the drug solution for the loading process. In a similar way, we loaded DOX, which is an anticancer drug, into the interior voids of the hollow particles to demonstrate the convenient encapsulation of small molecular drugs (Figure 3D,E). The encapsulation of the drug was also confirmed using DSC characterization. As shown in Figure 3F, the hollow NPs encapsulated with DOX exhibited a sharp peak at 213 °C that corresponded to the drug, indicating the successful encapsulation of DOX in the NPs.

**Figure 4:**
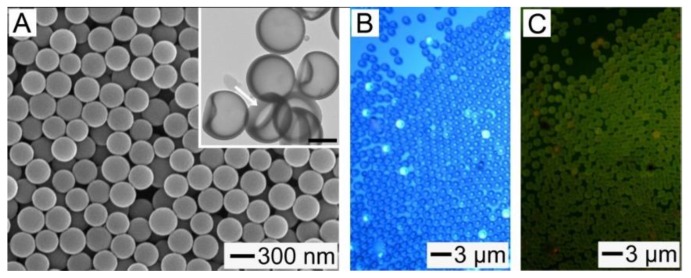
(**A**) SEM and TEM (inset) images of the hollow NPs containing the FA mixture and indocyanine green (ICG) in their shell. The scale bar in the inset represents 200 nm. (**B**) Optical and (**C**) fluorescence micrographs of the ICG-loaded hollow particles with *d_outer_* of 1.2 μm.

In order to provide the hollow NPs with a responsiveness to NIR light, we added them with ICG, which is a NIR light absorbing photothermal agent [21]. Its inclusion was accomplished through a series of processes involving the formation of a composite layer consisting of the FA mixture, PCL, and hydrophobic ICG-tetrabutylamine, the generation of corresponding composite rings, their conversion into hollow particles with openings on the shells, and the sealing of the openings. The SEM and TEM (inset) images in Figure 4A show the NPs fabricated from a mixture solution containing PCL (0.2 wt%), eutectic FA mixture (0.02 wt%), and hydrophobic ICG (0.0004 wt%), demonstrating that they had hollow voids and closed shells. Their mean outer diameter, obtained from these images, was 313 ± 18 nm, which is similar to the result measured by DLS (326 ± 23 nm), and they had a *ξ*-potential of −30.3 mV. The loading content of the FA mixture in the hollow NPs was 7.1%, which was consistent with the result for a similar sample without the photothermal agent (Figure 2A). As for the result in Figure 2B, this hollow structure allowed the NPs to be mechanically deformed, as marked with a white arrow in the inset. These results collectively suggest that the inclusion of ICG did not disturb the formation of the hollow NPs. Figure 4B,C shows the optical micrographs of the ICG-loaded hollow particles with closed shells. The fluorescence from ICG was observed in all the particles, which suggests its successful inclusion in each hollow particle.

**Figure 5:**
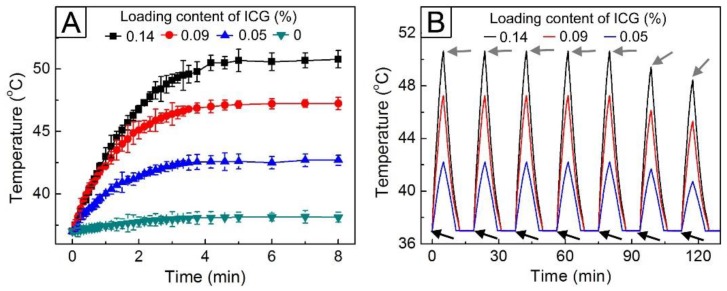
Time-dependent temperature curves of the suspensions of hollow NPs containing different amounts of ICG under (**A**) continuous and (**B**) on–off switched irradiation of 0.7 W/cm^2^ near-infrared (NIR) light. The black and gray arrows indicate the points of NIR light switching on and off, respectively.

The inclusion of ICG in the hollow NPs was further verified by investigating their photothermal ability. Figure 5A shows the time-dependent temperature curves for 5 mL of an aqueous suspension containing 5 mg of the hollow NPs under 0.7 W/cm^2^ NIR light. The sample without the inclusion of ICG exhibited no change in temperature, whereas the temperature of the samples containing ICG readily increased in response to NIR light irradiation because of the photothermal effect by ICG itself. This increase in temperature was strongly dependent on the loading content of ICG. The temperature of the NPs with a loading content of 0.05% (Figure 4A) was raised up to 42.5 °C. As the loading content increased to 0.14%, the temperature rise was even larger. The change in temperature could occur in an on–off manner when the NIR light was applied in an on–off way (Figure 5B). This reversible manner in temperature change was maintained when the light was switched on and off five times under the same condition. However, the temperature increase after the fifth cycle was not as large as that in the previous runs, which was due to the photobleaching of ICG [35,36]. Based on these results, the NPs with an ICG loading content of 0.05 wt% were used to further investigate the NIR light-triggered drug release.

**Figure 6:**
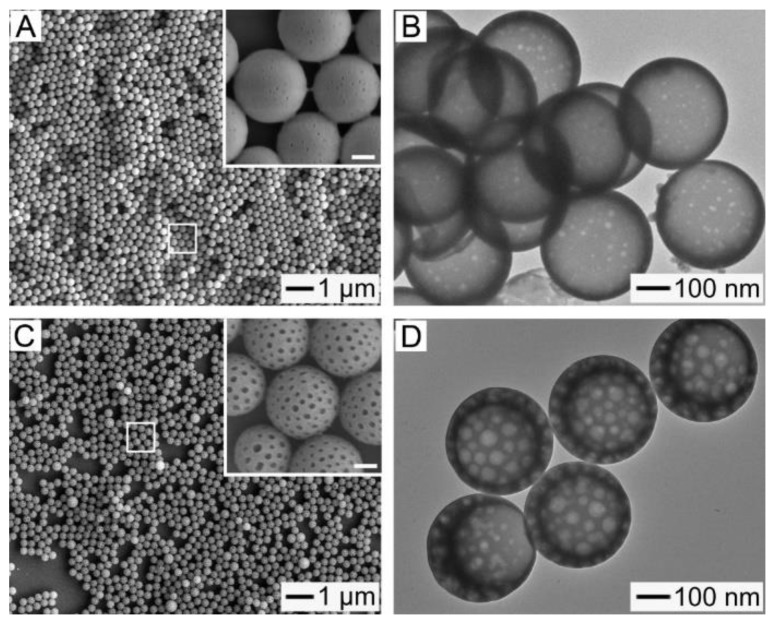
(**A**) SEM and (**B**) TEM images of the hollow NPs after exposing the sample with a loading content of 7.1% for the FA mixture to NIR light (0.7 W/cm^2^, 5 min). (**C**) SEM and (**D**) TEM images of the hollow NPs after exposing the sample with a loading content of 14.1% for the FA mixture to NIR light (0.7 W/cm^2^, 5 min). The insets are the magnified views of the areas marked with the white boxes, and their scale bars represent 100 nm.

Figure 6A shows an SEM image of the resultant NPs after treating the sample in Figure 4A with 0.7 W/cm^2^ NIR light. It is clear that they were still spherical without any change in size. The inset is a magnified view of the area marked by the white box and demonstrates the formation of nanopores with a diameter of ca. 15 nm on the shell of each NP, as further confirmed by the TEM image shown in Figure 6B. Under NIR light irradiation, the photothermal conversion by ICG included in the hollow NPs increased their local temperature above the melting point of the embedded FA mixture, leading to the melting away of the FA mixture and thereby the formation of nanopores on the shells of the NPs. The size of these pores could be increased by raising the amount of FA mixture in the hollow NPs. By using a mixture solution of PCL (0.2 wt%), FA mixture (0.04 wt%), and hydrophobic ICG (0.0004 wt%), we could obtain hollow NPs with a higher loading content (14.1%) of the FA mixture. Figure 6C shows an SEM image of the sample obtained after exposing the NPs to 0.7 W/cm^2^ NIR light. The image indicates that the resultant particles still had a spherical structure and a uniform size after the NIR treatment. The magnified view in the inset clearly demonstrates the formation of nanopores with a diameter of ca. 40 nm. However, despite the formation of these larger pores, the hollow interiors of the NPs were maintained without any destruction (Figure 6D).

**Figure 7:**
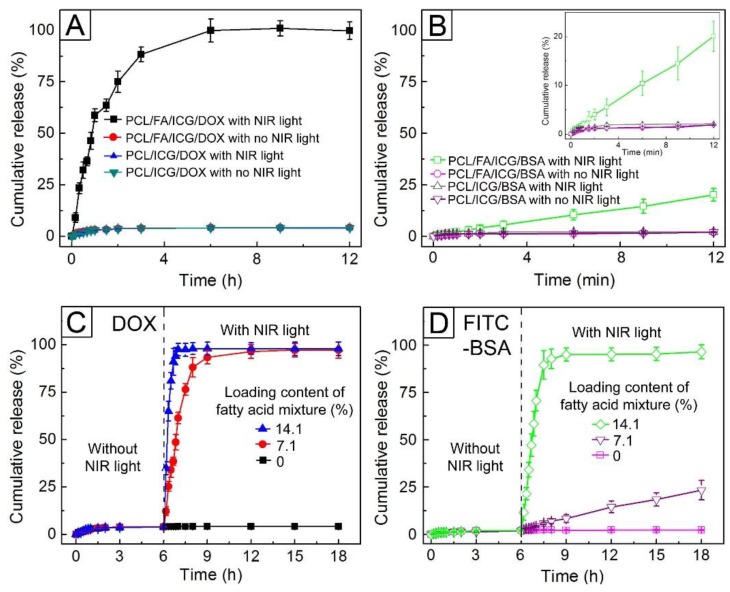
(**A**) Release profiles of DOX from PCL/ICG/DOX NPs and PCL/FA/ICG/DOX NPs with and without NIR light treatment (0.7 W/cm^2^, 5 min). (**B**) Release profiles of FITC-BSA from PCL/ICG/BSA NPs and PCL/FA/ICG/BSA NPs with and without NIR light treatment (0.7 W/cm^2^, 5 min). The inset shows the enlarged profiles. (**C**,**D**) Release profiles from the hollow NPs with different loading contents of the FA mixture for: (**C**) DOX and (**D**) FITC-BSA.

In order to investigate the NIR light-sensitive release behavior of DOX from the NPs encapsulating the drug, we prepared and tested the four types of hollow NPs: poly(ε-caprolactone) (PCL)/DOX NPs, PCL/ICG/DOX NPs, PCL/FA/DOX NPs, and PCL/FA/ICG/DOX NPs. The diameter, *ξ*-potential, and composition for each sample are summarized in Table S1 (Supplementary Materials), indicating that these aspects were similar for all the samples. Figure 7A shows the release profiles of DOX from PCL/FA/ICG/DOX NPs and PCL/ICG/DOX NPs. Without NIR light irradiation, these two samples released only 4.2% of the drug molecules over 12 h. On the other hand, when 0.7 W/cm^2^ NIR light was applied, their DOX release behaviors differed from each other, although the heat generation by ICG included in the NPs raised both their temperature up to 42.5 °C. The former allowed 100% of the drug to be released over 6 h, whereas the latter achieved a DOX release of 4.1% over the same period, similarly to the case without NIR light irradiation. Any change in particle size, which can affect the release behavior, was not observed during the release test. Thus, we attributed the NIR light-triggered release exhibited by the PCL/FA/ICG/DOX NPs to the nanopores formed by the melting away of the FA mixture, as shown in Figure 6. To support this conclusion, we investigated the release behaviors of DOX from PCL/DOX NPs and PCL/FA/DOX NPs, which could not form nanopores on their shells. Regardless of the NIR light irradiation, the two samples released approximately 4% DOX (Figure S5A, Supplementary Materials). These results suggest that the pores generated by NIR light functioned as effective channels for the quick release of the encapsulated drug molecules. 

We also investigated the release behaviors of FITC-BSA from the four types of hollow NPs, whose diameters, *ξ*-potential values, and compositions are summarized in Table S2 (Supplementary Materials). As for the case of DOX, only the PCL/FA/ICG/BSA NPs exhibited NIR light-triggered release of the protein (Figure 7B and Figure S5B). However, the release of the protein was much slower than that of DOX, which was attributed to the large size of the protein molecules. It has been reported that the protein molecule has a dimension of 14 nm × 4 nm × 4 nm or 9 nm × 5.5 nm × 5.5 nm [37,38]. Assuming that the molecule was spherical and had the same volume as a rectangular block, its diameter was calculated to be ca. 7.6 nm. The value is half the diameter of the formed nanopores (Figure 6A), suggesting a long time required for the macromolecules to be released through them. 

Figure 7C shows the release profiles of DOX from PCL/FA/ICG/DOX NPs with loading contents of 7.1% and 14.1% for the FA mixture. All the samples released approximately 4% of the drug molecules during the first 6 h without NIR light treatment. On the other hand, 0.7 W/cm^2^ NIR light irradiation led to the instant release of DOX from the hollow NPs, and the release profiles were strongly dependent on the loading content of the FA mixture. The NPs with a loading content of 7.1% achieved 100% release of the drug in 6 h after the NIR irradiation, whereas the ones with a loading content of 14.1% achieved the same result at only 1.5 h since the NIR treatment, which was due to the formation of the larger pores on the shells, as shown in Figure 6C. The effect of the pore size on the release behavior was more distinctly observed in the systems encapsulating FITC-BSA (Figure 7D). Without NIR light irradiation, the protein was not released from any of the types of NPs tested. On the contrary, after NIR light irradiation, the NPs with a loading content of 7.1% released 20% of the protein for 12 h since the NIR irradiation, and those with a loading content of 14.1% attained 100% release within 3 h of the NIR treatment, because their pores were large enough to let the protein molecules through.

**Figure 8:**
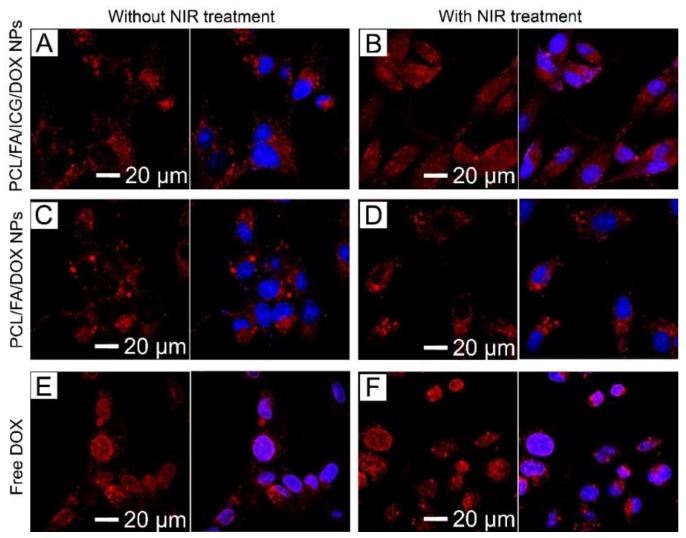
(**A**–**F**) Confocal laser scanning (CLS) micrographs showing the intracellular localization of DOX from PCL/FA/ICG/DOX NPs, PCL/FA/DOX NPs, free DOX internalized by SK-BR3 cells with and without NIR light irradiation (0.7 W/cm^2^, 8 min): (**A**) PCL/FA/ICG/DOX NPs without the NIR treatment, (**B**) PCL/FA/ICG/DOX NPs with the NIR treatment, (**C**) PCL/FA/DOX NPs without the NIR treatment, (**D**) PCL/FA/DOX NPs with the NIR treatment, (**E**) free DOX without the NIR treatment, and (**F**) free DOX with the NIR treatment. The left panels exhibit the images of DOX (red) in the cells, while the right panels show the merged images of DOX (red) and 4′,6-diamidino-2-phenylindole (DAPI)-counterstained nuclei of the cells (blue). The concentration of DOX was adjusted to 2 μg/mL for all the samples.

The CLS micrographs shown in Figure 8 demonstrate the intracellular localization of DOX from PCL/FA/ICG/DOX NPs, PCL/FA/DOX NPs, and free DOX internalized by human breast cancer SK-BR3 cells. The PCL/FA/ICG/DOX NPs were exclusively localized in the cytoplasm, as represented by the strong red fluorescence (Figure 8A). A similar result was observed in the cells treated with PCL/FA/DOX NPs (Figure 8C). However, the cells treated with free DOX mainly exhibited the strong red fluorescence in the nuclei (blue fluorescence) of the cells, as shown in Figure 8E, due to its diffusion through the cell membrane [39]. These results suggest that the NPs were internalized by endocytosis, and the drug molecules were encapsulated in the hollow voids of the NPs without their undesired release. When 0.7 W/cm^2^ NIR light was irradiated, a noticeable increase in red fluorescence was observed in the nuclei for the cells treated with PCL/FA/ICG/DOX NPs (Figure 8B), whereas the cells treated with PCL/FA/DOX NPs or free DOX did not exhibit such an increase (Figure 8D,F). This NIR light-triggered intranuclear release of the tested anticancer drug suggests that the presented NIR light-sensitive hollow NPs could act as drug-carrier candidates for on-demand cancer therapy. 

We evaluated the anticancer activity of the fabricated NIR light-sensitive hollow NPs via the WST-1 assay. The cytotoxicities of the hydrophobic ICG and PCL/FA NPs were preliminarily investigated. We did not observe significant changes in the viabilities of the SK-BR3 and NHDF cells in all the cases, relative to the control, where the NPs were not added (Figure S6, Supplementary Materials). In the case of the hydrophobic ICG, both types of the cells exhibited the viabilities above 90% up to a concentration of 1 mg/mL, compared with the control with no addition of the photothermal agent (Figure S7, Supplementary Materials). These results suggest the good biocompatibility of the NIR light-sensitive hollow NPs. Moreover, the cytotoxicity of NIR light was also tested, and the result confirmed that it was negligible (Figure S8, Supplementary Materials).

**Figure 9:**
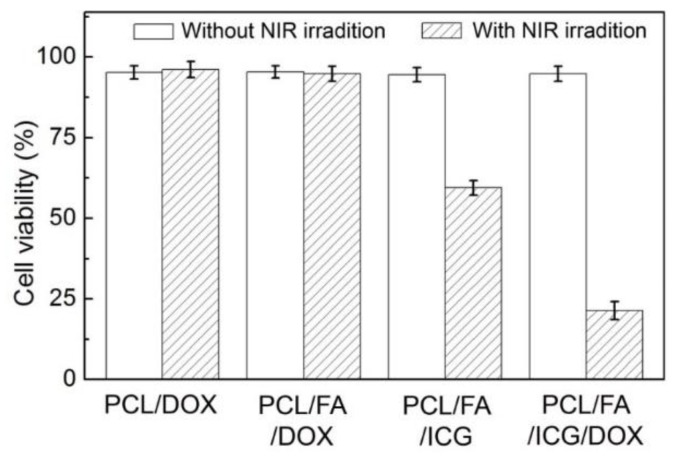
Viability of the SK-BR-3 cells treated with PCL/DOX NPs, PCL/FA/DOX NPs, PCL/FA/ICG NPs, and PCL/FA/ICG/DOX NPs without and with NIR light irradiation (0.7 W/cm^2^, 5 min).

Figure 9 shows the viabilities of SK-BR-3 cells treated with the four types of hollow NPs. Without NIR light irradiation, all the samples exhibited the viabilities above 90%. Such high values for the samples encapsulated with DOX (PCL/DOX NPs, PCL/FA/DOX NPs, and PCL/FA/ICG/DOX NPs) confirm the stable encapsulation of the drug without its undesired release from the NPs, which was consistent with the results of the in vitro release test shown in Figure 7. On the other hand, after 0.7 W/cm^2^ NIR light irradiation, the cell viability was significantly reduced only for the samples treated with the ICG-entrapped samples (PCL/FA/ICG NPs and PCL/FA/ICG/DOX NPs). In the case of the PCL/FA/ICG NPs, the cell viability decreased to 59% because of the hyperthermia effect by the entrapped ICG in response to the NIR light irradiation, while the PCL/FA/ICG/DOX NPs exhibited even more improved anticancer activity under NIR irradiation (viability of 21%). This improvement could be explained by the accumulation of DOX in the nuclei of cells (Figure 8B), where the drug molecules exerted their anticancer effect by intercalating with DNA [40]. These results imply that the NIR light-sensitive hollow NPs can have the feasibility of noninvasive, spatiotemporal control of drug release for cancer therapy, in conjunction with the NIR light-triggered photothermal effect.

## 4. Conclusions

We have demonstrated the fabrication biocompatible and biodegradable hollow NPs for the easy loading and NIR light-triggered release of drugs. The use of soft lithography allowed the generation of an array of discrete composite rings made of biocompatible and biodegradable PCL, biodegradable fatty acid with phase-change ability, and biocompatible ICG as the photothermal agent. These rings could be transformed into spherical hollow NPs with openings on the shells, which included the FA mixture and ICG. The openings functioned as a direct path for the loading macromolecules (BSA) as well as small molecular drugs (DOX) into the hollow interiors of the NPs, which could be closed for a stable encapsulation of the payloads. When irradiated with an external NIR source, the photothermal conversion by the entrapped ICG raised the local temperature of the hollow NPs above the melting point of the FA mixture, leading to the formation of surface nanopores, and hence the instant release of the encapsulated molecules through them. This NIR light-triggered release allowed the efficient intranuclear accumulation of DOX, resulting in excellent anticancer activity combined with the hyperthermia effect by the photothermal agent. The results suggested that the hollow NPs with NIR light sensitivity are potentially useful in the noninvasive, spatiotemporal control of anticancer drug release for targeted cancer therapy, in conjunction with the NIR light-driven photothermal effect, though further in vitro studies (cellular uptake mechanism and rate depending on their size and composition, their surface properties, and cell types), in vivo studies (selectivity and efficacy in targeting, biodistribution, and immune response), and clinical trials are required. Use of the hollow NPs that are capable of an easy loading of macromolecules is applicable to the controlled release of proteins. For biomacromolecular drugs, such as peptides and nucleic acids, that can be crucial in treating various types of cancers, further studies are needed. These studies are currently underway, and the results will be reported in the future.

## Supplementary Materials

The following are available online at https://www.mdpi.com/1999-4923/11/10/528/s1. Optical micrographs of a composite layer consisting of PCL and the FA mixture on top of a PVP layer; prediction of the diameter of the solid nanospheres prepared from the composite rings; scheme of a model for calculating the interfacial free energy of a hollow particle; SEM and TEM images of the micron-sized hollow particles for imaging by optical microscope; mean diameters, *ξ*-potential values, and compositions for the hollow particles encapsulating DOX and FITC-BSA; release profiles of DOX and FITC from PCL/DOX NPs and PCL/FA/DOX NPs; and viabilities of SK-BR-3 and NHDF cells treated with PCL/FA NPs, hydrophobic ICG, and NIR light.
