# Supplementary material for: Poly(ε-caprolactone) (PCL) Hollow Nanoparticles with Surface Sealability and On-Demand Pore Generability for Easy Loading and NIR Light-Triggered Release of Drug
Source: Pharmaceutics. 2019 Oct 13;11(10):528. doi: 10.3390/pharmaceutics11100528 (PMC6835703; doi:10.3390/pharmaceutics11100528)
Supplement: Supplementary file 1 [file pharmaceutics-11-00528-s001.pdf]

pharmaceutics

Supplementary Materials: Poly(ε-caprolactone) (PCL) Hollow Nanoparticles with Surface Sealability and On-Demand Pore Generability for Easy Loading and NIR Light-Triggered Release of Drug

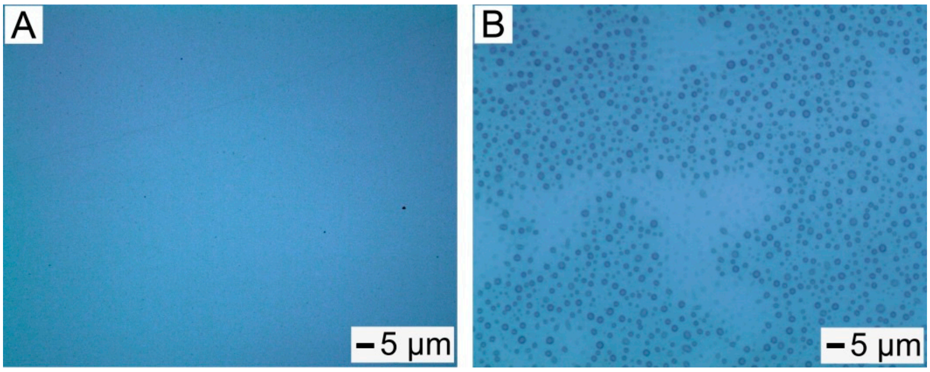

Figure S1. Optical micrographs of a composite layer consisting of PCL and the FA mixture on top of a PVP layer: (**A**) before and (**B**) after thermal annealing at 80 °C for 10 min.

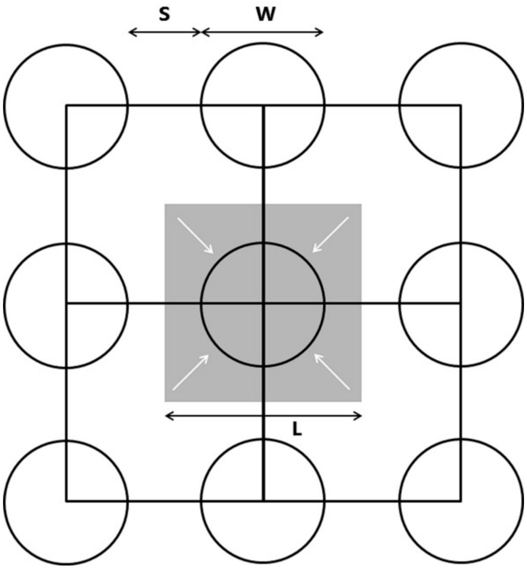

Figure S2. Scheme for prediction of the diameter of solid particles prepared from composite rings. Capillary force drives the composite into the recessed region on the mold, as indicated by white arrows, generating a discrete composite ring. Assuming that the volume of the composite layer is conserved during the molding process, the volume of each ring will be $V_{ring} = L^2t = At$, where A is the area, L is the side length of the area, and t is the thickness of the composite layer. L is the sum of w (the diameter of a cylindrical well on the poly(dimethyl siloxane) (PDMS) mold: 1 μm) and s (the separation distance between adjacent wells: 0.6 μm). For the composite layer with a thickness of 4.1 nm, V_{ring} is calculated to be 0.01 μm^3. Assuming each ring is transformed to one solid spherical particle, the diameter (d) of the particle can be calculated from the following equation: $V_{ring} = \pi(4/3)(d/2)^3$. The calculated value is 270 nm, which is in good agreement with the measured result (284 ± 12 nm).

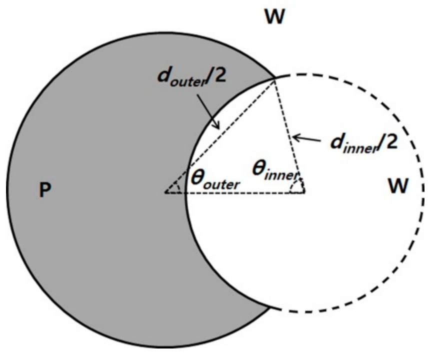

Figure S3. Model for calculating the interfacial free energy (*E*) of a hollow particle. *P* and *W* denote polymer and surrounding water phases, respectively.

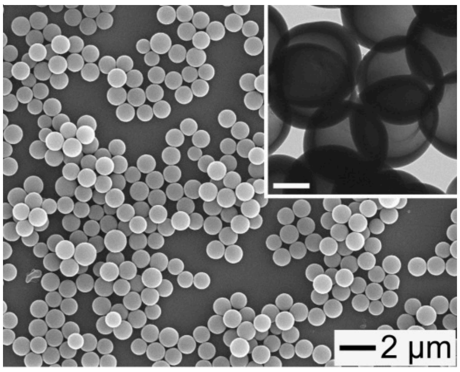

Figure S4. SEM and TEM (inset) images of the hollow particles with a closed shell, which were prepared from a composite layer of the FA mixture and PCL with a thickness of 105 nm. The layer was obtained from a mixture solution containing 0.5 wt% of the FA mixture and 5 wt% of PCL. The scale bar in the inset represents 500 nm.

Table S1. Mean diameters, ξ-potential values, and compositions for the four types of the hollow NPs encapsulating DOX. DLS: dynamic light-scattering.

Sample	Diameter (nm)		ξ-potential (mV)	Composition (wt%)			
	SEM/TEM	DLS		PCL	FA	ICG	DOX
PCL/DOX	297 ± 13	304 ± 19	–29.8	95.58	0	0	4.42
PCL/ICG/DOX	302 ± 17	315 ± 17	–30.1	95.54	0	0.05	4.41
PCL/FA/DOX	310 ± 15	321 ± 25	–30.8	88.52	7.11	0	4.37
PCL/FA/ICG/DOX	313 ± 18	326 ± 23	–30.5	88.37	7.14	0.05	4.44

Table S2. Mean diameters, ξ-potential values, and compositions for the four types of hollow NPs encapsulating FITC-BSA.

Sample	Diameter (nm)		ξ-potential	Composition (wt%)			
	SEM/TEM	DLS		PCL	FA	ICG	FITC-BSA
PCL/BSA	299 ± 15	305 ± 22	–30.5	97.82	0	0	2.18
PCL/ICG/BSA	300 ± 12	317 ± 23	–30.9	97.74	0	0.05	2.21
PCL/FA/BSA	311 ± 19	319 ± 22	–31.3	90.57	7.21	0	2.22
PCL/FA/ICG/BSA	312 ± 13	328 ± 26	–31.2	90.61	7.13	0.05	2.21

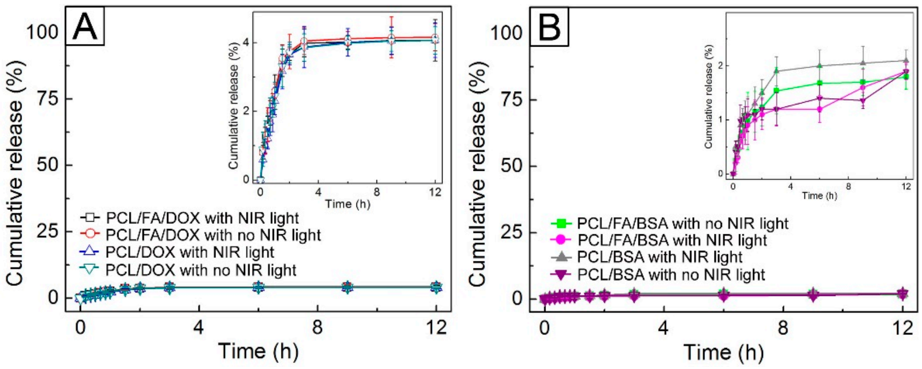

Figure S5. (**A**) Release profiles of DOX from PCL/DOX NPs and PCL/FA/DOX NPs with and without NIR light treatment (0.7 W/cm², 5 min). (**B**) Release profiles for FITC-BSA from PCL/BSA NPs and PCL/FA/BSA NPs with and without NIR light treatment (0.7 W/cm², 5 min). The insets show the enlarged profiles.

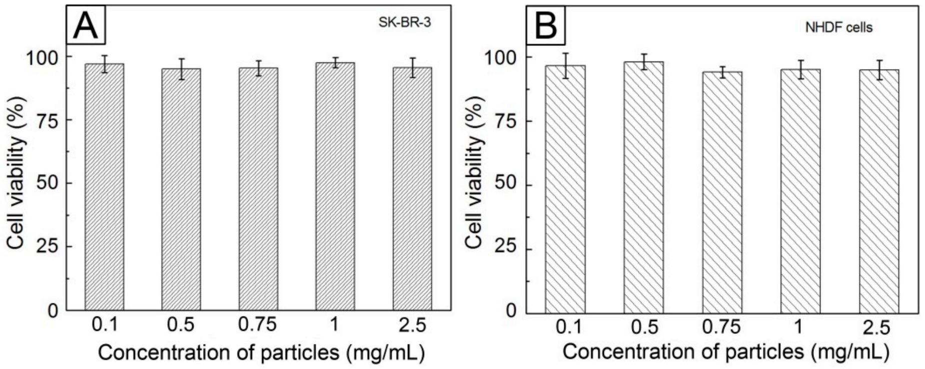

Figure S6. Viabilities of cells treated with PCL/FA NPs at various concentrations: (**A**) SK-BR-3 cells and (**B**) normal human dermal fibroblast (NHDF) cells.

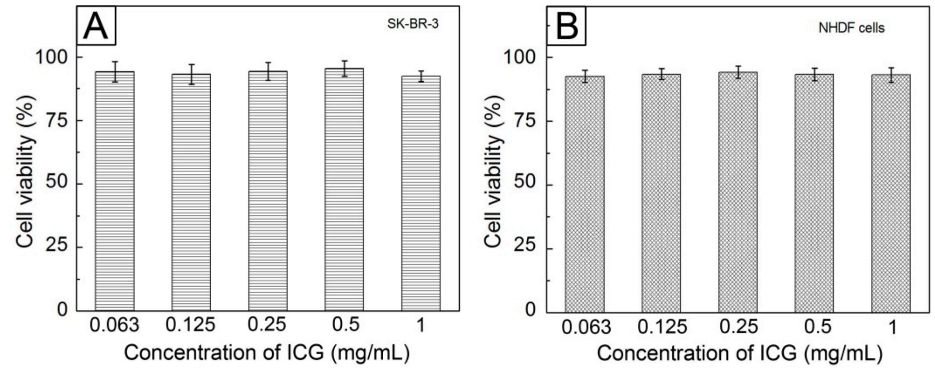

Figure S7. Viabilities of cells treated only with hydrophobic ICG: (**A**) SK-BR-3 cells and (B) NHDF cells.

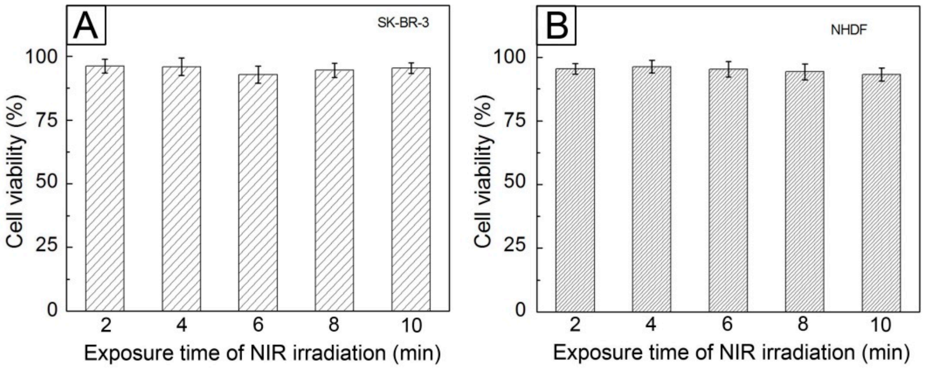

Figure S8. Viabilities of cells treated only with 0.7 W/cm^2 NIR irradiation: (**A**) SK-BR-3 cells and (**B**) NHDF cells.
